# Intermittently scanned continuous glucose monitoring compared with blood glucose monitoring is associated with lower HbA_1c_ and a reduced risk of hospitalisation for diabetes-related complications in adults with type 2 diabetes on insulin therapies

**DOI:** 10.1007/s00125-024-06289-z

**Published:** 2024-10-26

**Authors:** David Nathanson, Katarina Eeg-Olofsson, Tim Spelman, Erik Bülow, Mattias Kyhlstedt, Fleur Levrat-Guillen, Jan Bolinder

**Affiliations:** 1 Department of Medicine, Karolinska University Hospital Huddinge, Karolinska Institute, Stockholm, Sweden; 2 Medical Unit Endocrinology, Karolinska University Hospital Huddinge, Stockholm, Sweden; 3 Sahlgrenska University Hospital and Department of Molecular & Clinical Medicine, University of Gothenburg, Gothenburg, Sweden; 4 Centre of Registries Västra Götaland region, Gothenburg, Sweden; 5 Synergus RWE AB, Åkersperga, Sweden; 6 Abbott Laboratories Ltd, Maidenhead, UK

**Keywords:** Cardiovascular disease, CGM, Hospitalisation, Insulin treated, Intermittently scanned, Swedish National Diabetes Register, Swedish National Patient Register, Type 2 diabetes

## Abstract

**Aims/hypothesis:**

We assessed the impact of initiating intermittently scanned continuous glucose monitoring (isCGM) compared with capillary blood glucose monitoring (BGM) on HbA_1c_ levels and hospitalisations for diabetes-related complications in adults with insulin-treated type 2 diabetes in Sweden.

**Methods:**

This retrospective comparative cohort study included adults with type 2 diabetes who had a National Diabetes Register initiation date for isCGM after 1 June 2017. Prescribed Drug Register records identified subgroups treated with multiple daily insulin injections (T2D-MDI) or basal insulin (T2D-B), with or without other glucose-lowering drugs. The National Patient Register provided data on hospitalisation rates.

**Results:**

We identified 2876 adults in the T2D-MDI group and 2292 in the T2D-B group with an isCGM index date after 1 June 2017, matched with 33,584 and 43,424 BGM control participants, respectively. The baseline-adjusted difference in the change in mean HbA_1c_ for isCGM users vs BGM control participants in the T2D-MDI cohort was −3.7 mmol/mol (−0.34%) at 6 months, and this was maintained at 24 months. The baseline-adjusted difference in the change in HbA_1c_ for isCGM users vs BGM control participants in the T2D-B cohort was −3.5 mmol/mol (−0.32%) at 6 months, and this was also maintained at 24 months. Compared with BGM control participants, isCGM users in the T2D-MDI cohort had a significantly lower RR of admission for severe hypoglycaemia (0.51; 95% CI 0.27, 0.95), stroke (0.54; 95% CI 0.39, 0.73), acute non-fatal myocardial infarction (0.75; 95% CI 0.57, 0.99) or hospitalisation for any reason (0.84; 95% CI 0.77, 0.90). isCGM users in the T2D-B cohort had a lower RR of admission for heart failure (0.63; 95% CI 0.46, 0.87) or hospitalisation for any reason (0.76; 95% CI 0.69, 0.84).

**Conclusions/interpretation:**

This study shows that Swedish adults with type 2 diabetes on insulin who are using isCGM have a significantly reduced HbA_1c_ and fewer hospital admissions for diabetes-related complications compared with BGM control participants.

**Graphical Abstract:**

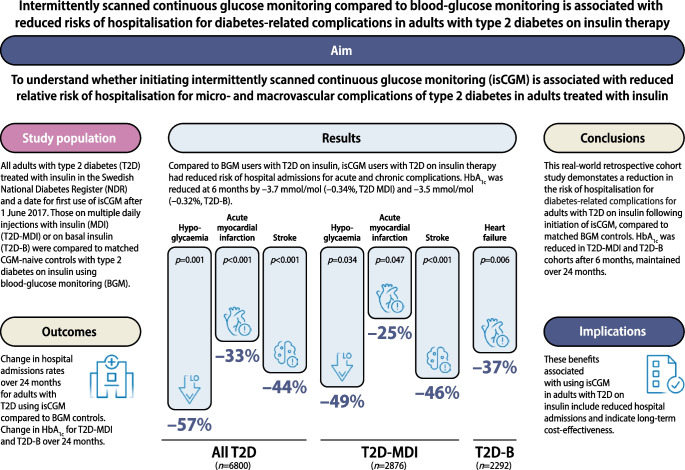

**Supplementary Information:**

The online version contains peer-reviewed but unedited supplementary material available at 10.1007/s00125-024-06289-z.


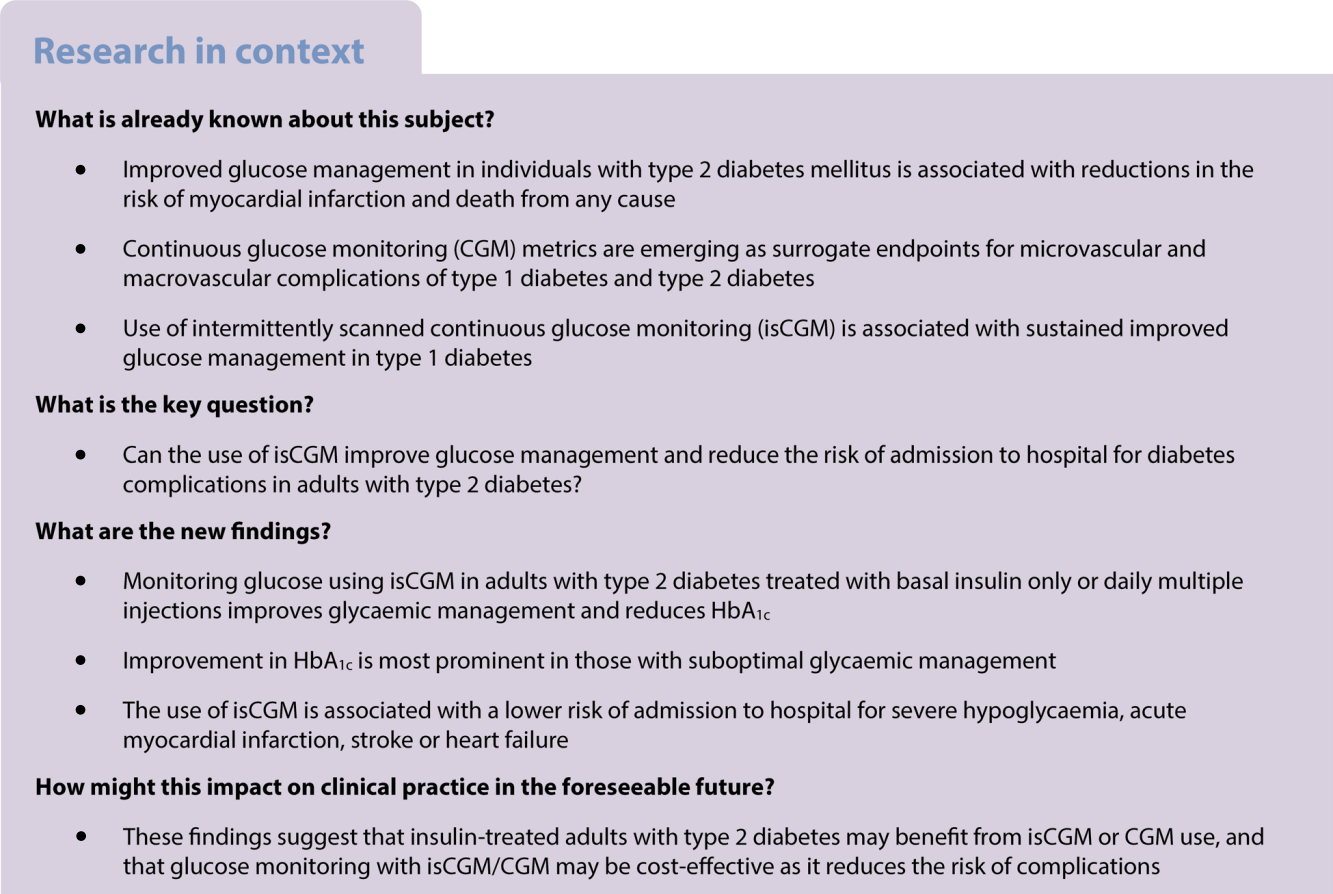


## Introduction

The UK Prospective Diabetes Study (UKPDS) study has shown that intensive glucose management, starting at the time of diagnosis of type 2 diabetes, is associated with sustained reductions in the risk of myocardial infarction and death from any cause, in addition to reductions in the risk of microvascular disease [1–3]. RCTs and prospective real-world studies have shown that use of continuous glucose monitoring (CGM) or intermittently scanned continuous glucose monitoring (isCGM) in people with type 2 diabetes on intensive insulin therapy through multiple daily injections is associated with lower HbA_1c_ [4–6] and reductions in hypoglycaemia [7, 8]. Likewise, RCTs and retrospective studies have also demonstrated that using these sensor-based glucose monitoring systems is associated with improvements in HbA_1c_ for people with inadequately managed type 2 diabetes treated with basal insulin only [9–11] or on non-insulin therapies [12–14].

Together with reductions in HbA_1c_ and hypoglycaemia, CGM-derived metrics are now emerging as surrogate endpoints for microvascular and macrovascular complications of type 1 and type 2 diabetes [15]. For people with type 2 diabetes, improved CGM metrics for time in range are associated with a decreased risk of retinopathy [16, 17] and renal disease [18]. In terms of macrovascular outcomes, lower time in range and glycaemic variability are associated with risk markers for CVD in type 2 diabetes [19–22], and lower time in range in people with type 2 diabetes is associated with an increased risk of all-cause and CVD mortality [23], as well as peripheral artery disease [24].

Although these observations may suggest that microvascular and macrovascular events leading to hospitalisation should decrease for people with type 2 diabetes using CGM, no studies, to our knowledge, have investigated in greater detail the rate of hospital admissions for diabetes-related complications in people with type 2 diabetes following initiation of CGM as part of their glycaemic management.

In Sweden, approximately 5.0% of the entire population are recorded in the National Diabetes Register (NDR) as having type 2 diabetes, with a registry coverage of 90% [25]. The NDR has been an integral part of diabetes care in Sweden since 1996, and registration of people with diabetes in Sweden is encouraged at least once per year. The goal of the NDR is to monitor and improve diabetes care, as well as to reduce diabetes-related morbidity and mortality. As part of this mission, the NDR facilitates comparisons between a range of treatment modalities and clinical outcome measures. From June 2016 onwards, the NDR has documented use of CGM devices among adults with diabetes, including isCGM.

In the current study, using the unique ten-digit personal identity number assigned to permanent residents in Sweden [26], we linked data from the NDR on adults with type 2 diabetes who were recorded as isCGM users with data on those using conventional capillary blood glucose monitoring (BGM). The information from the NDR was then linked with data from the Swedish Prescribed Drug Register (SPDR) [27], which contains individualised data on prescribed drugs, to identify subgroups of people with type 2 diabetes treated with multiple daily insulin injections (T2D-MDI) or on basal insulin (T2D-B), with or without non-insulin glucose-lowering drugs. These data were then cross-linked with the Swedish National Patient Register (NPR) of hospital admissions [28].

The aims of this study were to assess the impact of initiating isCGM compared with BGM on longitudinal changes in HbA_1c_ from baseline for adults in Sweden with type 2 diabetes on intensive or non-intensive insulin therapy, and to investigate the occurrence of severe hypoglycaemia and the rates of hospital admission for known microvascular and macrovascular complications of diabetes and overall hospitalisation for any reason.

## Methods

This comparative retrospective cohort study used data extracted from the NDR and included adults aged ≥18 years with type 2 diabetes with a diabetes clinic visit recorded in the NDR after 1 January 2014 and up to 22 August 2022, and a first recorded use (index date) of isCGM from 1 June 2017 onwards. Exclusion criteria were prior use of CGM or isCGM systems. Sex, gender and ethnicity are recorded in the NDR at the person level. However, no selection was undertaken based on these or other characteristics and all participants with type 2 diabetes meeting the inclusion criteria were included in the analysis. The type 2 diabetes diagnosis was based on a clinician’s diagnosis in primary care or hospital-based diabetes outpatient clinics. A propensity score-weighted BGM control group was identified, comprising adults with type 2 diabetes who continued to use BGM testing only (see below).

### Data completeness

New incident users of isCGM registered in the NDR were assessed for two variables: HbA_1c_ values and prior use of isCGM. Data were collected in line with international consensus standards on HbA_1c_ reporting in mmol/mol, and converted into % units according to the International Federation of Clinical Chemistry (IFCC) reference system for national standardisation [29, 30]. Missing values in each of these categories occur if the information is unknown, or if the assessment was not conducted or recorded by the responsible healthcare professional.

### Incident use of isCGM in individuals with type 2 diabetes

**Fig. 1:**
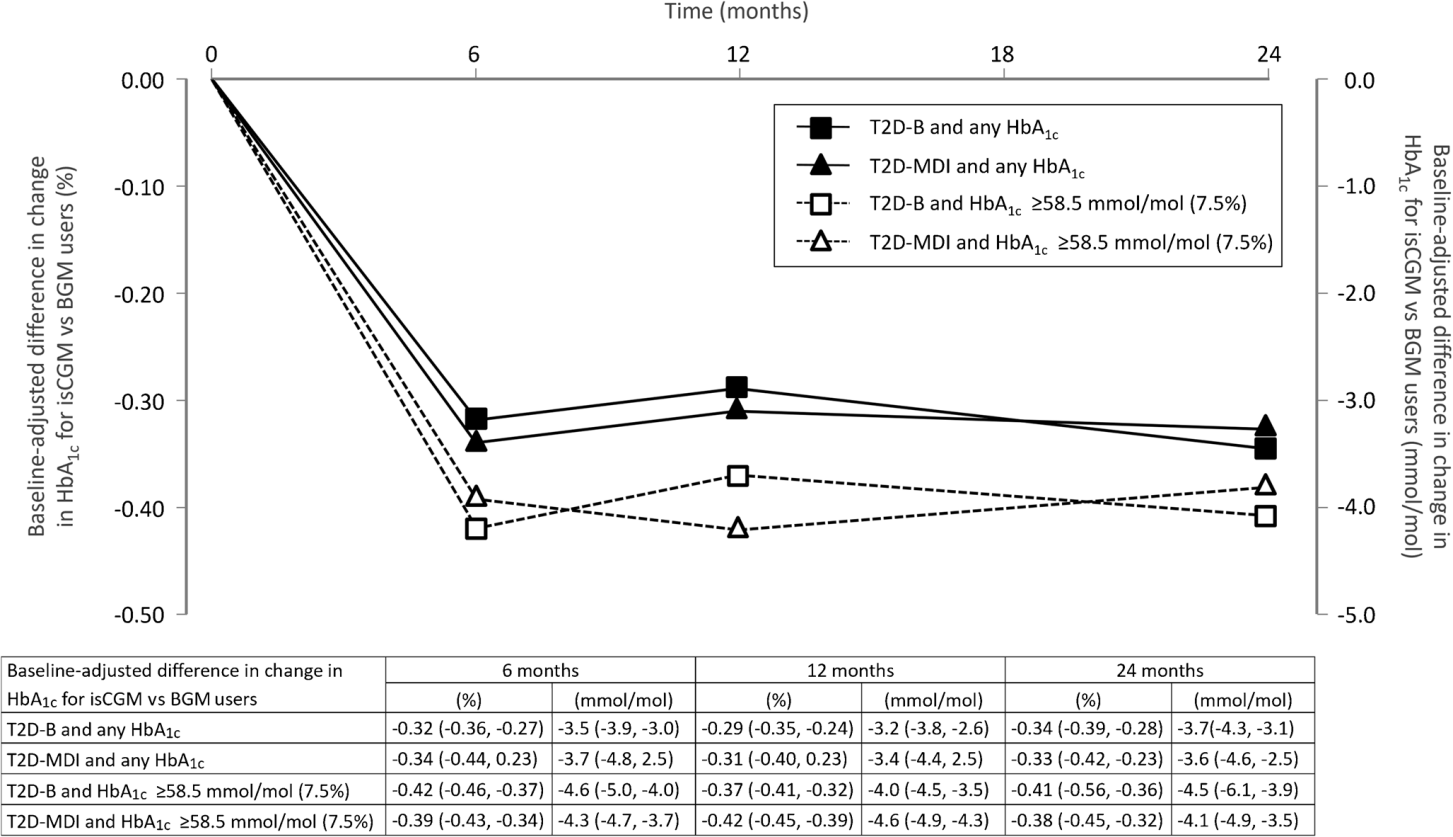
Baseline-adjusted differences in change in HbA_1c_ for isCGM users vs BGM control participants with type 2 diabetes treated with either basal insulin only (T2D-B) or with multiple daily injections with insulin (T2D-MDI). Data are shown for isCGM users vs HbA_1c_-matched BGM control participants on comparable insulin regimens with any HbA_1c_ and for those with baseline HbA_1c_ ≥58 mmol/mol (≥7.5%). Change from baseline was significant for all observations (*p*<0.001). Values in the table are the baseline-adjusted differences in the change in HbA_1c_ (with 95% CIs) for isCGM users vs BGM control participants. PS-IPTW mean regression was used to compare changes in HbA_1c_ between isCGM users and control participants

All individuals with type 2 diabetes and an NDR index date for first use of isCGM between 1 June 2017 and 22 August 2022 were identified within each calendar year. The identification and selection process for new incident isCGM users and BGM control participants is described in electronic supplementary material (ESM) Fig. 1. This study is focused on new incident users of isCGM within the 6, 12 and 24 months following their index date.

### Assessing change in HbA_1c_ among incident isCGM users vs BGM control participants

HbA_1c_ is a recorded variable for people with type 2 diabetes in the NDR, and is typically recorded at least once a year. We compared the most recent laboratory-measured HbA_1c_ value recorded within the 3–14 month period prior to the index date vs the HbA_1c_ values recorded at three timepoints: the day between day 91 and day 272 after the index date that was closest to the 6-month timepoint (day 181.5), the day between day 273 and day 455 after the index date that was closest to the 12-month timepoint (day 363.5), and the day between day 456 and day 818 after the index date that was closest to the 24-month timepoint (day 727). HbA_1c_ measurements were available within the defined before and after periods for a subset of the total study population who were incident isCGM users. Using these criteria, the change in HbA_1c_ was evaluated for all incident users with type 2 diabetes treated with insulin based on the baseline HbA_1c_ prior to the index date and the longitudinal HbA_1c_ values at 6, 12 and 24 months after the index date. Data for the change in HbA_1c_ are presented as baseline-adjusted differences in the change in HbA_1c_ for incident isCGM users vs the control groups of BGM users.

### Differentiating insulin treatment among incident isCGM users vs BGM control participants

An objective assessment of insulin treatment status for new incident isCGM users and BGM control participants was obtained using retrospective pharmacy dispensing data recorded in the SPDR [27], which provides nationwide data on all prescribed medicines dispensed at all Swedish pharmacies, including the Anatomical Therapeutic Chemical classification codes, as well as the date of prescription and dispensing. Included individuals were linked to SPDR records to identify subgroups treated either with multiple daily insulin injections (T2D-MDI) or on basal insulin only (T2D-B), with or without other glucose-lowering drugs in each case.

### Hospital admission rates for incident isCGM users and BGM control participants

Hospital admission data for new incident isCGM users and BGM control participants were extracted from the Swedish NPR [28] which provides administrative data on inpatient care, including hospital admissions, with associated diagnoses and procedures coded according to ICD-10 (https://icd.who.int/browse10/2019/en) (ESM Table 1).

### Statistical analysis

Categorical variables were summarised using frequency and percentage. Continuous variables were summarised using the mean ± SD or the median (IQR), as appropriate. Propensity score-based inverse probability of treatment weighting (PS-IPTW) linear regression, adjusted for baseline HbA_1c_, was used to compare changes in HbA_1c_ between isCGM users and control participants using BGM. A PS-IPTW negative binomial regression was used to compare rates of hospital admission for diabetes-related events between isCGM users and control participants, offset by follow-up duration. The propensity score was derived using a logistic regression in which isCGM/BGM usage status was the binary outcome, and age, sex, BMI, baseline HbA_1c_, lipid profile, renal function, smoking status, physical activity, pre-baseline comorbidity and diabetic complications were the independent explanatory variables (see ESM Table 2). The performance of the weighting was assessed through derivation and analysis of weighted standardised differences. A double robust method, using additional variable adjustment in the regression analysis, was used to minimise the effect of remaining imbalance after weighting [31]. Our approach was to include any variable that remained associated with an absolute weighted standardised difference of 15% as an additional explanatory variable in the final modelling. Hospital admission counts were assessed for overdispersion. For all analyses, a *p* value <0.05 was considered significant. All analyses were performed using Stata version 17 (StataCorp, USA) and R version 4.2.3 (R Foundation for Statistical Computing, Austria).

### Ethical approval

The study protocol has been approved by the Swedish Ethical Review Authority (Dnr 2021-02886).

## Results

We identified 6800 adults with type 2 diabetes on any therapy who had at least one registration of isCGM use during the study period (Table 1 and ESM Fig. 1) and for whom HbA_1c_ was recorded within 3–14 months prior to the index date and at the 6-, 12- and 24-month longitudinal timepoints specified. Of these, 2876 (42.3%; 37.3% female) were in the T2D-MDI cohort and 2292 (33.7%; 38.3% female) were in the T2D-B cohort; 1632 individuals with type 2 diabetes with at least one registration of isCGM were not on either multiple daily injections or basal insulin therapy and were not included in the analysis. The baseline characteristics of the T2D-MDI and T2D-B groups are shown in Table 1, together with the characteristics of 33,584 and 43,424 control participants (out of a total of 78,386) with T2D treated with multiple daily insulin injections or basal insulin, respectively, who used BGM testing only. Although the mean diabetes duration was similar in the total cohorts of isCGM users and BGM control participants (almost 16 years, Table 1), the isCGM group had a lower mean baseline age than the BGM control group (63.50 years vs 70.11 years, Table 1). Mean HbA_1c_ was similar between the isCGM and BGM groups, and evidence of macrovascular complications at baseline was more prevalent in the isCGM group (ESM Table 2). After PS-IPTW, the magnitude (absolute value) for 16 of the 19 estimated weighted standardised differences was <0.1, a threshold that is taken to indicate a negligible difference in the mean or prevalence of a covariate between treatment groups [32]. This negligible level of difference applied to all baseline characteristics between the isCGM and BGM groups, including existing diabetes complications (ESM Table 2), except mean age, diastolic BP and eGFR at baseline. The weighted standardised differences for these three characteristics were each <0.2, which is the common threshold for small differences [33], and is considered unlikely to have any meaningful effect on the outcomes.

**Table 1:** Baseline characteristics of incident isCGM users and BGM control groups with type 2 diabetes

	All participants			T2D-MDI cohort			T2D-B cohort		
	isCGM (*n*=6800)	BGM (*n*=78,386)	Weighted standardised difference	isCGM (*n*=2876)	BGM (*n*=33,584)	Weighted standardised difference	isCGM (*n*=2292)	BGM (*n*=43,424)	Weighted standardised difference
Age (years)	63.50 ± 12.70	70.11 ± 11.31	−0.148	62.79 ± 13.24	71.38 ± 10.91	−0.138	62.88 ± 12.42	69.12 ± 11.47	−0.114
Sex									
Female	2665 (39.2)	31,343 (40.0)	0.000	1073 (37.3)	13,446 (40.0)	−0.011	877 (38.3)	17,352 (40.0)	−0.000
Male	4135 (60.8)	47,043 (60.0)		1803 (62.7)	20,138 (60.0)		1415 (61.7)	26,072 (60.0)	
BMI (kg/m^2^)	30.14 ± 4.40	30.40 ± 4.39	−0.052	30.11 ± 4.33	30.67 ± 4.50	−0.05	30.46 ± 4.31	30.22 ± 4.30	−0.045
HbA_1c_ (mmol/mol)	61.97 ± 15.52	62.08 ± 13.44	−0.045	66.01 ± 15.08	59.45 ± 13.12	0.061	66.01 ± 16.07	62.62 ± 13.01	0.146
HbA_1c_ (%)	7.82 ± 1.42	7.83 ± 1.23		8.19 ± 1.38	7.59 ± 1.20		8.19 ± 1.47	7.88 ± 1.19	
Diabetes duration (years)	15.81 ± 9.87	15.72 ± 8.82	−0.013	18.95 ± 10.36	17.43 ± 9.12	0.067	16.18 ± 8.66	14.44 ± 8.32	0.050

Values are means ± SD or *n* (%)

### Longitudinal change in HbA_1c_ for isCGM users compared with BGM control participants

The overall baseline-adjusted difference in the change in mean HbA_1c_ in the T2D-MDI cohort for isCGM users vs BGM users was −3.7 mmol/mol (−0.34%) at 6 months, and this was maintained at 12 months (−3.4 mmol/mol or −0.31%) and at 24 months (−3.6 mmol/mol or −0.33%; *p*<0.001 in all instances) (Fig. 1). For isCGM users vs BGM users in the T2D-B cohort, the baseline-adjusted difference in the change in mean HbA_1c_ was −3.5 mmol/mol (−0.32%) at 6 months, −3.2 mmol/mol (−0.29%) at 12 months, and −3.7 mmol/mol (−0.34%) at 24 months (*p*<0.001 in all instances).

### Longitudinal change in HbA_1c_ for isCGM users with suboptimal glycaemic management

In both the T2D-B and T2D-MDI treatment cohorts, isCGM users with suboptimal glycaemic management and baseline HbA_1c_ ≥58.5 mmol/mol (≥7.5%) were compared with HbA_1c_-matched BGM control participants with type 2 diabetes on basal insulin and multiple daily injection regimens (Fig. 1). For isCGM users with type 2 diabetes and suboptimal glycaemic management at baseline, reductions in HbA_1c_ relative to BGM users were greater than in the cohorts with any HbA_1c_. In the T2D-MDI cohort, HbA_1c_ was reduced by 4.3 mmol/mol (0.39%) at 6 months, 4.6 mmol/mol (0.42%) at 12 months and 4.1 mmol/mol (0.38%) at 24 months (*p*<0.001 in all instances). For isCGM users vs BGM users in the T2D-B cohort, the change in mean HbA_1c_ was −4.6 mmol/mol (−0.42%) at 6 months, –4.0 mmol/mol (−0.37%) at 12 months, and −4.5 mmol/mol (−0.41%) at 24 months (*p*<0.001 in all instances).

In a further analysis, we analysed the mean change in HbA_1c_ from baseline to 6, 12 and 24 months after the isCGM index date for adults with type 2 diabetes and suboptimal glucose management, treated with MDI (T2D-MDI) or with basal insulin only (T2D-B), compared with prior use of BGM only (ESM Table 3). Reductions in HbA_1c_ from baseline after the isCGM index date were significant for individuals with HbA_1c_ ≥58.5 mmol/mol (≥7.5%), with similar reductions in the T2D-B (−8.0 mmol/mol, 0.73%; *p*<0.0001) and T2D-MDI (−8.3 mmol/mol, −0.76%; *p*<0.0001) cohorts at 6 months, and this was maintained at 24 months (−8.3 mmol/mol, −0.76% in both cohorts; *p*<0.0001). For adults with HbA_1c_ ≥70 mmol/mol (≥8.6%) prior to the index date, both the T2D-B cohort and the T2D-MDI cohorts achieved greater reductions in HbA_1c_ from baseline after starting isCGM, with a mean decrease of 14.3 mmol/mol (−1.31%) at 6 months for both cohorts (*p*<0.0001 in both cases), which was maintained at 24 months (ESM Table 3).

### Inpatient hospital admissions for adults after the isCGM index date relative to BGM control participants

Using data from the NPR, we calculated the RR for admission to hospital for a number of known diabetes complications as well as hospitalisation for any reason, after the isCGM index date, for all adults with type 2 diabetes (Table 2), and separately for the T2D-MDI cohort (Table 3) and the T2D-B cohort (Table 4).Following the isCGM index date, adults with type 2 diabetes using isCGM had lower RRs for hospital admission for hypoglycaemia (0.43; 95% CI 0.27, 0.70; *p*=0.001), stroke (0.56; 95% CI 0.46, 0.69; *p*<0.001), heart failure (0.63; 95% CI 0.53, 0.76; *p*<0.001), acute myocardial infarction (0.67; 95% CI 0.56, 0.82; *p*<0.001) and hospitalisation for any reason (0.71; 95% CI 0.67, 0.75; *p*<0.001) than BGM users. isCGM users in the T2D-MDI cohort had significantly lower RRs for hospital admission for severe hypoglycaemia (0.51; 95% CI 0.27, 0.95; *p*=0.034), stroke (0.54; 95% CI 0.39, 0.73; *p*<0.001), acute myocardial infarction (0.75; 95% CI 0.57, 0.99; *p*=0.047) or hospitalisation for any reason (0.84; 95% CI 0.77, 0.90, *p*<0.001). The RR for hospital admissions for isCGM users in the T2D-B cohort was reduced for heart failure (0.63; 95% CI 0.46, 0.87; *p*=0.006) and reduced for admissions for any reason (0.76; 95% CI 0.69, 0.84; *p*<0.001). The risks for hospital admission for angina were significantly increased in adults with type 2 diabetes overall and in the T2D-B cohort, and just missed being significant in the T2D-MDI cohort (*p*=0.051).

**Table 2:** Hospital admissions for diabetes complications for isCGM users with type 2 diabetes vs BGM control participants (all participants)

Primary diagnosis at hospital admission	No. of hospital admissions		Hospitalisation event rate per 100 person-years of follow-up (95% CI)		RR (95% CI)	*p* value
	isCGM (*n*=6800)	BGM (*n*=78,386)	isCGM (*n*=6800)	BGM (*n*=78,386)		
Hypoglycaemia	22	928	0.17 (0.10, 0.25)	0.33 (0.31, 0.35)	0.43 (0.27, 0.70)	0.001
Coma	1	14	0.01 (0.00, 0.04)	0.01 (0.00, 0.01)	Insufficient events	N/A
Acute myocardial infarction^a^	219	6174	1.65 (1.44, 1.89)	2.20 (2.15, 2.26)	0.67 (0.56, 0.82)	<0.001
Angina	181	2905	1.37 (1.18, 1.58)	1.04 (1.00, 1.08)	1.27 (1.03, 1.57)	0.025
Ischaemic heart disease	68	1327	0.51 (0.40, 0.65)	0.47 (0.45, 0.50)	1.08 (0.80, 1.44)	0.621
Stroke	174	5387	1.31 (1.13, 1.53)	1.92 (1.87, 1.97)	0.56 (0.46, 0.69)	<0.001
Peripheral vascular disease	16	323	0.12 (0.07, 0.20)	0.12 (0.10, 0.13)	0.98 (0.49, 1.96)	0.961
Heart failure	434	11,664	3.28 (2.98, 3.60)	4.16 (4.09, 4.24)	0.63 (0.53, 0.76)	<0.001
Atrial fibrillation	167	3237	1.26 (1.08, 1.47)	1.16 (1.12, 1.20)	1.00 (0.80, 1.25)	0.994
Kidney disease	485	9329	3.66 (3.35, 4.01)	3.33 (3.26, 3.40)	0.89 (0.77, 1.04)	0.144
Retinopathy	14	183	0.11 (0.06, 0.18)	0.07 (0.06, 0.08)	1.55 (0.81, 2.98)	0.189
Neuropathy	2	40	0.02 (0.00, 0.05)	0.01 (0.00, 0.02)	1.05 (0.21, 5.38)	0.951
Foot ulcer	4	114	0.03 (0.00, 0.08)	0.04 (0.03, 0.05)	0.71 (0.24, 2.10)	0.533
Hospitalisation for any reason^b^	7930	183,170	59.91 (58.59, 61.24)	65.34 (65.04, 65.64)	0.71 (0.67, 0.75)	<0.001

PS-IPTW negative binomial regression was used to calculate rates of hospital admission for diabetes-related events per 100 person-years of cumulative follow-up. RRs (95% CIs) are for admission to hospital for isCGM users compared with BGM control participants

^a^Non-fatal

^b^Any reason not limited to the renal and cardiovascular events listed above

N/A, not applicable

**Table 3:** Hospital admissions for diabetes complications for isCGM users in the T2D-MDI cohort vs BGM control participants

	No. of hospital admissions		Hospitalisation event rate per 100 person-years of follow-up (95% CI)		RR (95% CI)	*p* value
Primary diagnosis at hospital admission	isCGM (*n*=2876)	BGM (*n*=33,584)	isCGM (*n*=2876)	BGM (*n*=33,584)		
Hypoglycaemia	14	510	0.27 (0.15, 0.45)	0.44 (0.40, 0.48)	0.51 (0.27, 0.95)	0.034
Coma	1	6	0.02 (0.00, 0.11)	0.01 (0.00, 0.01)	Insufficient events	N/A
Acute myocardial infarction^a^	109	2900	2.10 (1.72, 2.53)	2.50 (2.41, 2.60)	0.75 (0.57, 0.99)	0.047
Angina	87	1352	1.67 (1.34, 2.06)	1.17 (1.11, 1.23)	1.37 (1.00, 1.87)	0.051
Ischaemic heart disease	39	617	0.75 (0.53, 1.03)	0.53 (0.49, 0.58)	1.36 (0.91, 2.05)	0.132
Stroke	77	2398	1.48 (1.17, 1.85)	2.07 (1.99, 2.15)	0.54 (0.39, 0.73)	<0.001
Peripheral vascular disease	11	164	0.21 (0.11, 0.38)	0.14 (0.12, 0.16)	1.42 (0.59, 3.46)	0.435
Heart failure	258	5634	4.96 (4.38, 5.61)	4.86 (4.74, 4.99)	0.86 (0.68, 1.10)	0.242
Atrial fibrillation	74	1466	1.42 (1.12, 1.79)	1.27 (1.20, 1.33)	1.03 (0.74, 1.43)	0.880
Kidney disease	297	4507	5.71 (5.08, 6.40)	3.89 (3.78, 4.01)	1.18 (0.95, 1.46)	0.133
Retinopathy	8	102	0.15 (0.07, 0.30)	0.09 (0.07, 0.011)	1.66 (0.66, 4.18)	0.280
Neuropathy	0	17	0.00 (0.00, 0.07)	0.01 (0.00, 0.02)	Insufficient events	N/A
Foot ulcer	4	53	0.08 (0.02, 0.20)	0.05 (0.03, 0.06)	1.57 (0.45, 5.46)	0.476
Hospitalisation for any reason^b^	4248	85,849	81.71 (79.27, 84.21)	74.09 (73.59, 74.58)	0.84 (0.77, 0.90)	<0.001

PS-IPTW negative binomial regression was used to calculate rates of hospital admission for diabetes-related events per 100 person-years of cumulative follow-up. RRs (95% CIs) are for admission to hospital for isCGM users compared with BGM control participants

^a^Non-fatal

^b^Any reason not limited to the renal and cardiovascular events listed above

N/A, not applicable

**Table 4:** Hospital admissions for diabetes complications for isCGM users in the T2D-B cohort vs BGM control participants

	No. of hospital admissions		Hospitalisation event rate per 100 person-years of follow-up (95% CI)		RR (95% CI)	*p* value
Primary diagnosis at hospital admission	isCGM (*n*=2292)	BGM (*n*=43,424)	isCGM (*n*=2292)	BGM (*n*=43,424)		
Hypoglycaemia	8	405	0.18 (0.08, 0.36)	0.25 (0.23, 0.28)	0.69 (0.31, 1.44)	0.305
Coma	0	7	0.00 (0.00, 0.08)	0.00 (0.00, 0.01)	Insufficient events	N/A
Acute myocardial infarction^a^	86	3175	1.96 (1.57, 2.42)	1.99 (1.92, 2.06)	0.92 (0.67, 1.27)	0.622
Angina	74	1494	1.69 (1.32, 2.12)	0.93 (0.88, 0.98)	1.75 (1.24, 2.47)	0.002
Ischaemic heart disease	23	683	0.52 (0.33, 0.79)	0.43 (0.40, 0.46)	1.28 (0.77, 2.12)	0.337
Stroke	68	22,892	1.55 (1.20, 1.96)	1.81 (1.74, 1.88)	0.74 (0.52, 1.03)	0.075
Peripheral vascular disease	2	157	0.05 (0.00, 0.16)	0.10 (0.08, 0.11)	0.42 (0.08, 2.08)	0.288
Heart failure	131	5838	2.98 (2.49, 3.54)	3.65 (3.56, 3.75)	0.63 (0.46, 0.87)	0.006
Atrial fibrillation	47	1,713	1.07 (0.79, 1.42)	1.07 (1.02, 1.12)	0.92 (0.62, 1.38)	0.694
Kidney disease	125	4662	2.85 (2.37, 3.39)	2.92 (2.83, 3.00)	0.79 (0.59, 1.05)	0.105
Retinopathy	4	75	0.09 (0.02, 0.23)	0.05 (0.04, 0.06)	1.93 (0.65, 5.72)	0.235
Neuropathy	1	22	0.02 (0.00, 0.13)	0.01 (0.00, 0.02)	1.69 (0.12, 23.78)	0.699
Foot ulcer	0	57	0.00 (0.00, 0.08)	0.04 (0.03, 0.05)	Insufficient events	N/A
Hospitalisation for any reason^b^	2462	93,900	56.06 (53.87, 58.32)	58.75 (58.37, 59.12)	0.76 (0.69, 0.84)	<0.001

PS-IPTW negative binomial regression was used to calculate rates of hospital admission for diabetes-related events per 100 person-years of cumulative follow-up. RRs (95% CIs) are for admission to hospital for isCGM users compared with BGM control participants

^a^Non-fatal

^b^Any reason not limited to the renal and cardiovascular events listed above

N/A, not applicable

## Discussion

In this retrospective study, we have linked data from the Swedish NDR with records from other national healthcare registries and performed propensity score-weighted analyses to compare large cohorts of adults with type 2 diabetes on insulin therapies who self-manage their glucose levels using either isCGM or BGM.

The first observation of note is that isCGM users with type 2 diabetes on insulin therapy achieve comparable and sustained reductions in HbA_1c_ after initiating isCGM, irrespective of treatment with either intensive multiple daily injections or basal insulin-only regimens. Compared with control groups using BGM, both the T2D-MDI cohort and the T2D-B cohort demonstrated HbA_1c_ reductions of 3.7 mmol/mol (0.34%) and 3.5 mmol/mol (0.32%), respectively, 6 months after starting isCGM, and this was maintained for 24 months in both cohorts (Fig. 1). These data support earlier studies showing that use of isCGM in people with type 2 diabetes is associated with lower HbA_1c_, whether they are treated with multiple daily injections [5, 6], basal insulin only [10, 11] or any glucose-lowering therapies [34]. Notably, in absolute terms, the improvements in HbA_1c_ for isCGM users recorded in our study are also almost identical to those observed in previous RCTs in which the efficacy of real-time CGM vs BGM on glucose management was assessed in individuals with type 2 diabetes treated with multiple daily injections [4] or basal insulin only [9]. Moreover, for isCGM users with suboptimal glycaemic management and baseline HbA_1c_ ≥58.5 mmol/mol (≥7.5%), reductions in HbA_1c_ were higher than for the overall population of isCGM users with type 2 diabetes on basal insulin or multiple daily injections (Fig. 1 and ESM Table 3). For isCGM users in the T2D-MDI and T2D-B cohorts with HbA_1c_ ≥70 mmol/mol (≥8.6%), reductions in HbA_1c_ from baseline were even greater (ESM Table 3), with reductions in HbA_1c_ of 14.3 mmol/mol (1.31%) for both cohorts after 6 months, which were sustained at 24 months. Again, the reductions in HbA_1c_ were comparable between the T2D-B and T2D-MDI cohorts for individuals with suboptimal glycaemic management.

The second clinically important observation relates to our analysis of the hospital admissions data (Tables 2, 3 and 4), which showed that the entire group using isCGM (T2D-MDI and T2D-B) had significantly lower risk of hospitalisation for severe hypoglycaemia (−57%), stroke (−44%), heart failure (−37%) and acute non-fatal myocardial infarction (−33%) relative to BGM control participants (Table 2). Risk of hospitalisation for any reason was also significantly reduced in the whole isCGM user group (−29%) (Table 2), and in both the T2D-MDI and the T2D-B subgroups using isCGM, compared with the BGM control groups (−16% and −24%, respectively) (Tables 3 and 4). These outcomes are comparable with similar reductions in hospital admission rates for any cause reported in two studies of people with type 2 diabetes, one after initiating isCGM [35] and the other using any CGM method [36].

However, the multiple daily injection and basal insulin-only regimens showed different risk profiles for hospitalisation for known complications of diabetes. First, the data demonstrated a reduction in hospitalisation for severe hypoglycaemia only in the T2D-MDI cohort (−49%, *p*=0.034) but not in the T2D-B cohort (Tables 3 and 4). In the RELIEF study, using the French national health claims database, hospitalisation for hypoglycaemia over the 2 year study period was reduced both for people with type 2 diabetes on any therapy (43% fewer admissions), of whom 85% were on intensive insulin therapy [37], but also for the smaller subgroup with type 2 diabetes on basal insulin only (44% fewer admissions) [38]. This difference in results may reflect a more conservative approach to using the different insulin regimens in type 2 diabetes in Sweden compared with France, as the ratio of T2D-MDI and T2D-B therapies was more equal in our study, and the hospital event rates for hypoglycaemia in the T2D-B control cohort (0.25 per 100 person-years, Table 4) were lower than those reported in the RELIEF study cohort for the 12 months prior to starting isCGM (0.73% of study population). Nonetheless, the comparable reductions in admission rates for severe hypoglycaemia in people with type 2 diabetes on multiple daily injections seen in both studies clearly suggest a preventive effect of isCGM on the acknowledged increased risks of severe hypoglycaemia for people with type 2 diabetes on intensive insulin therapy. In accordance with these results, another study reported a 29% reduction in hospital admissions for hypoglycaemia for people with type 2 diabetes on prandial insulin after starting isCGM [39]. To our knowledge, no studies have reported on admissions for severe hypoglycaemia in type 2 diabetes after commencing use of other CGM devices.

Second, regarding hospitalisation for major cardiovascular events, such as stroke, heart failure and non-fatal acute myocardial infarction, for adults with type 2 diabetes, we found reduced risk of admission for stroke and non-fatal acute myocardial infarction for adults in the T2D-MDI cohort (−46% and −25%, respectively) and a 37% reduced risk of admission for heart failure in the T2D-B cohort. While we believe our study is the first to report the impact of using isCGM or other CGM technologies on alleviating the risks for hospitalisation for these macrovascular complications of type 2 diabetes, the underlying reasons for this favourable finding, and for the different outcomes in the intensive and non-intensive insulin treatment groups, remain to be elucidated. Major risk factors for CVD, such as lipids, BP and renal function, were adjusted for in the analysis and should not have had a bearing on the results. Although it cannot be completely ruled out, it seems unlikely that these risk reductions can be solely explained by the improvement in glycaemic management after initiating isCGM, given the rather modest difference in HbA_1c_ between the isCGM and BGM groups and the relatively short follow-up period. As the risk of major cardiovascular events for people with type 2 diabetes is associated with hypoglycaemia, including nocturnal and severe hypoglycaemia [40, 41], it may be proposed that the reduced risk of severe hypoglycaemia in adults with type 2 diabetes in Sweden who are using isCGM is associated with a reduced occurrence of major cardiovascular events. Other possible reasons for this reduced occurrence include an increase in time in range and reduced glucose variability [19–22]. However, this possibility requires further research and detailed analyses of CGM metrics, which were not available in this study.

### Strengths and limitations

It is important to acknowledge that our study has limitations. As use of isCGM is only one of a number of variables in our retrospective cohort study, it is possible that other factors may have influenced the observed outcomes. For example, the NDR data do not record whether the isCGM user received device training or education from their healthcare professionals around the index date, which may have resulted in improved diabetes self-care behaviours that are not controlled for in this retrospective analysis. Furthermore, the study does not contain data on duration of insulin use, which could have had an effect on HbA_1c_ reduction. However, the groups were well balanced after the PS-IPTW for all measured variables, with weighted standardised differences below 0.10, which indicates a negligible treatment group difference and is not considered to be confounding, or below 0.20, which indicates only small differences between the covariates, such that any residual confounding is unlikely to affect the outcomes in a meaningful way.

It must also be acknowledged that the validity of the outcomes from an investigation using a diabetes registry and a wider patient registry relies on the quality of interpretation and application of the variables within each of the registries. For example, our control group had an older mean age at baseline compared with the isCGM intervention group; however, the weighted standard difference between these two groups was below 0.2, which is indicative of only a small difference between these groups. Diabetes duration and HbA_1c_ were similar between the two groups, and the baseline prevalence of cardiovascular complications was similar or even higher in the isCGM users than in the BGM control participants, with weighted standard differences for all these covariates being lower than 0.1. Strengths of our study include the large population of people with type 2 diabetes on insulin therapies included, and the fact that the NDR, SPDR and NPR have greater than 90% coverage of all adults with diabetes in Sweden. Another strength is that specialised nurses provide education to people with type 2 diabetes before starting isCGM in Sweden, which ensures that use of the system is appropriate.

As the clinical type of diabetes that a person has is stored at the person level in the NDR and is updated to be the last recorded observation, there should be no error in the variable containing clinical diabetes type. Thus, interpretation of our study outcomes is not confounded by errors in classification of diabetes type. This means that selection bias should not be a factor. An important strength of our analysis is the inclusion of control groups, identified from within the NDR, that match the baseline characteristics of our study cohorts across a wide range of variables, but who were not incident users of isCGM or other CGM devices between June 2017 and August 2022. This has allowed us to control for selection bias and to mitigate the impact of other factors on glycaemic management over the study period, independent of isCGM.

### Conclusion

This real-world retrospective cohort study of adults with type 2 diabetes in Sweden, using analysis of data from the NDR linked with comprehensive prescribing information from the SPDR, has shown that adults with type 2 diabetes on intensive insulin therapy with multiple daily injections and adults with type 2 diabetes treated with basal insulin only achieve significant and comparable reductions in HbA_1c_ 6 months after being prescribed isCGM, and these reductions persist for at least 24 months. Furthermore, both in adults with type 2 diabetes treated with multiple daily injections and adults with type 2 diabetes treated with basal insulin, the reductions in HbA_1c_ are positively correlated with the HbA_1c_ at baseline.

In addition, we have been able to demonstrate a significantly lower risk of hospitalisation for severe hypoglycaemia or for major cardiovascular events, or hospitalisation for any reason, for adults with type 2 diabetes following initiation of use of the isCGM system compared with control participants who are using BGM. These outcomes have direct consequences for optimising health-related outcomes for people with type 2 diabetes, and have implications for the long-term cost-effectiveness of providing access to isCGM for people with type 2 diabetes on insulin therapies.

## Supplementary information

Below is the link to the electronic supplementary material.

ESM (PDF 286 KB)

## Data Availability

Data not presented in the analysis are available on request from the authors.

## Abbreviations

BGM: Blood glucose monitoring
CGM: Continuous glucose monitoring
isCGM: Intermittently scanned continuous glucose monitoring
NDR: National Diabetes Register
NPR: National Patient Register
PS-IPTW: Propensity score-based inverse probability of treatment weighting
SPDR: Swedish Prescribed Drug Register
T2D-B: Subgroup of type 2 diabetes participants treated with basal insulin
T2D-MDI: Subgroup of type 2 diabetes participants treated with multiple daily insulin injections

## Authors’ relationships and activities

DN received honoraria from Abbott for the work within this study. KE-O has received fees for lecturing and/or honoraria for consulting from Sanofi, Novo Nordisk, Eli Lilly and Abbot Diabetes Care. TS has received compensation for serving on scientific advisory boards and honoraria for consultancy from Biogen, as well as speaker honoraria from Novartis. MK is employed by Synergus RWE, which received reimbursement from Abbott to perform the analysis. FL-G is an employee of Abbott Diabetes Care. JB has received honoraria for consulting and/or lecture fees from Abbott Diabetes Care, MannKind Corporation, Nanexa, Nordic InfuCare, Novo Nordisk and Sanofi.
