# Supplementary material for: Intermittently scanned continuous glucose monitoring compared with blood glucose monitoring is associated with lower HbA1c and a reduced risk of hospitalisation for diabetes-related complications in adults with type 2 diabetes on insulin therapies
Source: Diabetologia. 2024 Oct 26;68(1):41–51. doi: 10.1007/s00125-024-06289-z (PMC11663194; doi:10.1007/s00125-024-06289-z)
Supplement: Supplementary file 1 — ESM (PDF 286 KB) [file 125_2024_6289_MOESM1_ESM.pdf]

Electronic Supplementary Materials

Intermittently scanned glucose monitoring compared with blood glucose monitoring is associated with lower HbA_{1c} and reduced risk of hospitalisation for diabetes-related complications in adults with T2D on insulin therapies

David Nathanson[1,2], Katarina Eeg-Olofsson[3,4], Tim Spelman[5], Erik Bülow[4], Mattias Kyhlstedt[5], Fleur Levrat-Guillen[6], Jan Bolinder[1].

1. Department of Medicine, Karolinska University Hospital Huddinge, Karolinska Institute, Stockholm, Sweden
2. Medical Unit Endocrinology, Karolinska University Hospital Huddinge, Stockholm, Sweden
3. Sahlgrenska University Hospital and Department of Molecular & Clinical Medicine, University of Gothenburg, Sweden
4. Centre of Registries Västra Götaland region, Gothenburg, Sweden
5. Synergus RWE AB, Åkersperga, Sweden
6. Abbott Laboratories Ltd, Maidenhead, UK

ESM Table 1. Coding of diabetes, complications of diabetes, and concomitant disease for registry-based identification using ICD 10 codes and hospital procedure codes (KVÅ).

**Diabetes complications and concomitant conditions**	**ICD 10 codes or procedural codes**
Type 2 or unspecified diabetes	E11, E14
Angina pectoris	I20
Acute myocardial infarction	I21
Ischemic heart disease	I22-I25
Atrial fibrillation	I48
Heart failure	I50
Stroke	I61, I63, I64, I67.9
Peripheral vascular disease, PVD	I70.2, I73.1, I73.9, I79.2, E11.5, E14.5
Lower extremity amputation	KVÅ: NEQ19, NEQ99, NFQ09,<br>NFQ19, NFQ99,<br>NGQ09, NGQ19, NGQ99,<br>NHQ09, NHQ11, NHQ12, NHQ13, NHQ14, NHQ16, NHQ17, NHQ99
Other sudden death, cause unknown	R96.0, R96.1
Diabetic foot and ulcers	E11.6, E14.4, L00, L03, L08, L97
Eye disease and diabetic retinopathy	H0-H4, H50-H53, H55, H57-H59, E103, E113, E143<br>KVÅ: DT006
Vision loss or blindness on one or two eyes	H54
Symptomatic neuropathy	E11.4D, G62.9, G56, G58.9, G99, G63.2, G73.3, M14.6
Kidney disease	E11.2, E14.2<br>E11.7, E11.8, E14.7, E14.8, R809, N00-N08, N10-N19, N28.9
End-stage renal disease, ESRD, with dialysis or kidney transplantation	Dialysis Z49, Z94, Z99.2<br>KVÅ Dialysis DR015, DR016, DR017<br>KVÅ Transplantation KAS10, KAS20, VF420
Hyperglycaemia	R73.9
Type 2 diabetes with ketoacidosis	E11.1, 11.10 (without coma), 11.11 (with coma)
Hypoglycaemia without/with coma	E11.0C, E11.6, E11.6A, E14.0C, E14.6A, E16.0, E16.1, E16.2
Coma (unspecified)	E11.0, E14.0, R40.2

Note: KVÅ - Klassifikation av vårdåtgärder [Classification of health care interventions; NBHW]

ESM Table 2. Baseline factors that were included in the derivation of the propensity scores

Characteristic at baseline registration in dataset	Category	isCGM users (n=6,800)	BGM Controls (n=78,386)	Standardised difference	Weighted standardised difference*
Age (years) - mean (SD)		63.50 (12.70)	70.11 (11.31)	-0.549	-0.148
Sex - n (%)	Female	2665 (39.2)	31343 (40.0)	-0.016	0.000
	Male	4135 (60.8)	47043 (60.0)		
BMI - mean (SD)		30.14 (4.40)	30.40 (4.39)	-0.059	-0.052
HbA_{1c} - mean (SD)	mmol/mol	62.00 (15.50)	62.10 (13.40)	-0.012	-0.045
	%	7.82 (1.42)	7.83 (1.23)		
Diabetes duration (years) - mean (SD)		15.81 (9.87)	15.72 (8.82)	0.011	-0.013
SBP - mean (SD)	mmHg	134.39 (13.78)	134.97 (14.35)	-0.041	-0.021
DBP - mean (SD)	mmHg	76.85 (8.55)	74.95 (8.95)	0.217	0.146
LDL - mean (SD)	mmHg	2.45 (0.78)	2.41 (0.68)	0.058	0.055
HDL - mean (SD)	mmol/L	1.20 (0.30)	1.19 (0.27)	0.028	0.025
Triglycerides - mean (SD)	mmol/L	2.09 (1.45)	1.98 (0.98)	0.088	0.050
Total cholesterol - mean (SD)	mmol/L	4.32 (0.95)	4.32 (0.84)	-0.007	0.007
Creatinine - mean (SD)	µmol/L	98.33 (73.03)	94.38 (44.26)	0.065	0.028
eGFR - mean (SD)	mL/min/1.73m²	77.48 (28.58)	73.00 (23.90)	0.170	0.138
Albuminuria - n (%)	No	5215 (76.7)	60269 (76.9)	-0.002	0.017
	Previous	401 (5.9)	3565 (4.6)		
	Microalbuminuria	810 (11.9)	10851 (13.8)		
	Macroalbuminuria	374 (5.5)	3701 (4.7)		
Physical activity - n (%)	Never	1067 (15.7)	16039 (20.5)	-0.056	-0.058
	Less than once a week	1445 (21.3)	13226 (16.9)		
	Regularly - 1-2 times a week	1167 (17.2)	13870 (17.7)		
	Regularly - 3-5 times a week	1169 (17.2)	14704 (18.8)		
	Daily	1952 (28.7)	20547 (26.2)		
Ischaemic heart disease - n (%)		1929 (28.4)	20713 (26.4)	-0.044	-0.040
Retinopathy - n (%)		3327 (48.9)	37462 (47.8)	-0.023	-0.016
Stroke - n (%)		809 (11.9)	7886 (10.1)	-0.059	-0.050
Smoker - n (%)		950 (14.0)	9083 (11.6)	-0.071	-0.063

ESM Table 3. Change from baseline HbA_{1c} for adults with T2D treated with MDI or basal insulin and suboptimal glycaemic control after starting isCGM

Treatment population, stratified by HbA_{1c} at baseline	Mean Baseline HbA_{1c} % (SD)	Mean change from baseline at 6 months	p-value	Mean change from baseline at 12 months	p-value	Mean change from baseline at 24 months	p-value
All T2D-B	8.19 (1.47)	-0.37 (1.42)	<0.0001	-0.36 (1.48)	<0.0001	-0.40 (1.53)	<0.0001
Baseline HbA_{1c} ≥7.5%	8.86 (1.27)	-0.73 (1.47)	<0.0001	-0.75 (1.52)	<0.0001	-0.76 (1.55)	<0.0001
Baseline HbA_{1c} ≥8.6%	9.66 (1.10)	-1.31 (1.64)	<0.0001	-1.31 (1.66)	<0.0001	-1.37 (1.68)	<0.0001
All T2D-MDI	8.19 (1.38)	-0.37 (1.36)	<0.0001	-0.36 (1.38)	<0.0001	-0.37 (1.38)	<0.0001
Baseline HbA_{1c} ≥7.5%	8.74 (1.20)	-0.76 (1.39)	<0.0001	-0.76 (1.40)	<0.0001	-0.76 (1.43)	<0.0001
Baseline HbA_{1c} ≥8.6%	9.77 (1.21)	-1.31 (1.59)	<0.0001	-1.31 (1.60)	<0.0001	-1.38 (1.62)	<0.0001

Table shows mean change in HbA_{1c} from baseline for adults with T2DM treated with MDI (T2D-MDI) or with basal insulin only (T2D-B) at 6, 12 and 24 months after the isCGM index date. Data is included for all T2D-MDI and T2D-B, and for subgroups with suboptimal glycaemic control stratified by baseline HbA_{1c}. Data represent mean % change in HbA_{1c} (±SD) at 6, 12 and 24 months after starting isCGM compared to prior baseline using SMBG (see below).

Mean baseline indicates the most recent HbA_{1c} recorded in the NDR within 3-14 months prior to the isCGM index date. Change from baseline calculated from HbA_{1c} values recorded at three timepoints: day 91 – day 272 after the index date that was closest to the 6-month timepoint (day 181.5); between day 273– day 455 after the Index date that was closest to the 12 month timepoint (day 363.5), and; between day 456 – day 818 after the Index date that was closest to the 24 month timepoint (day 727).

NDR, National Diabetes Register; T2D, type 2 diabetes; T2D-MDI, type 2 diabetes treated with multiple daily injections with insulin; T2D-B, type 2 diabetes treated with basal insulin; SD, standard deviation.

ESM Figure 1. The identification and selection process for new incident isCGM users and BGM controls

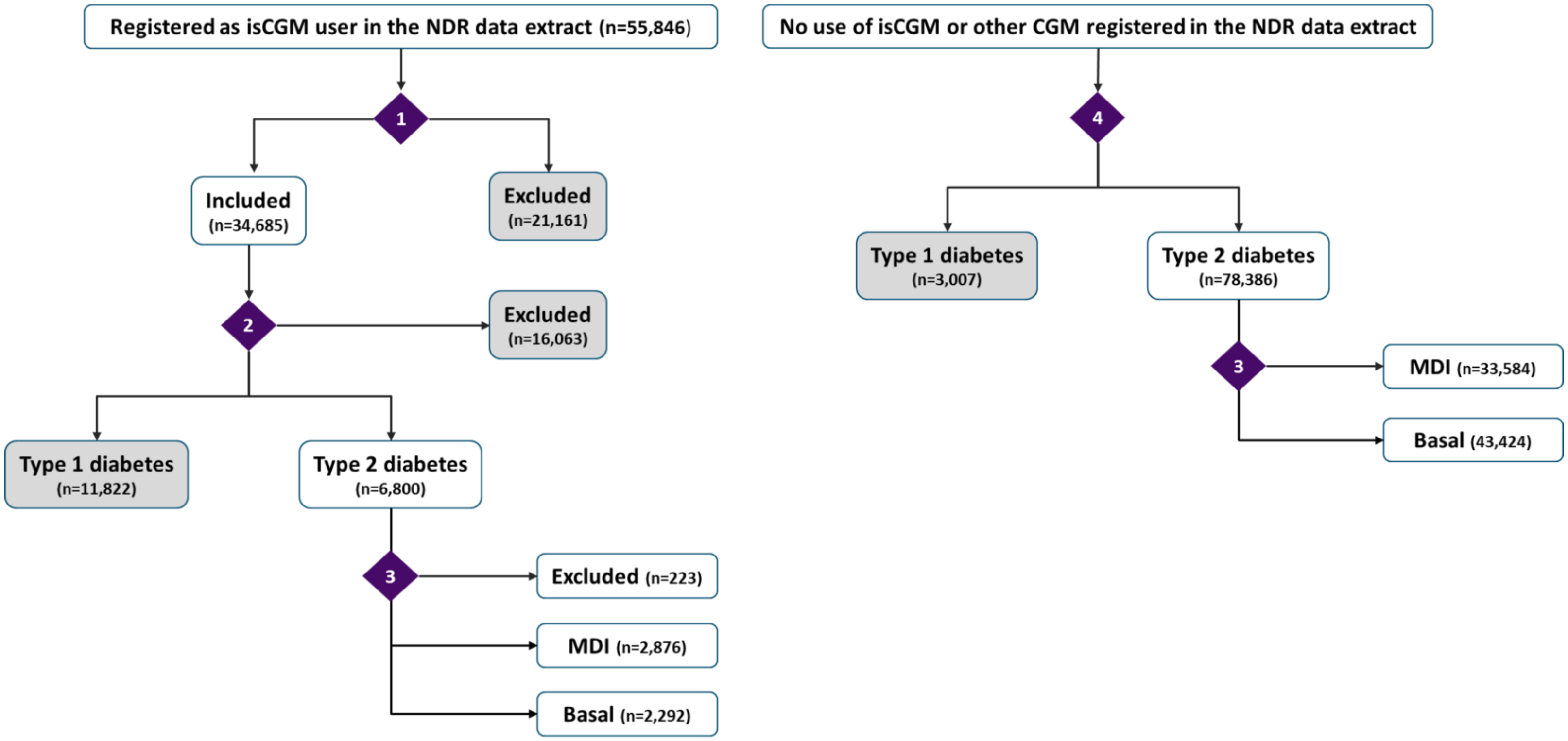

Selection points as numbered

**1**	First recorded exposure to FSL from 1st of June 2017 onwards<br>No prior use of other CCM<br>Minimum data set for analysis	**3**	Derivation rules for treatment sub-group
**2**	Type 1: HbA_{1c} baseline value 3-8 months prior to index date<br>Type 2: HbA_{1c} baseline value 3-14 months prior to index date.	**4**	Matched to isCGM group using logistic regression for independent explanatory variables, see Supplementary Table 1

BGM, blood glucose monitoring; isCGM, intermittently-scanned continuous glucose monitoring; MDI, multiple daily injections with insulin; NDR, National Diabetes Register
